# Valorization of date palm by-products *(Phoenix dactylifera L.)* in cake fortification: Nutritional enrichment, antioxidant retention, and shelf-life extension

**DOI:** 10.1016/j.fochx.2025.103184

**Published:** 2025-10-17

**Authors:** Abdelrahman R. Ahmed, Khaled M.A. Ramadan, Haiam O. Elkatry, Nashi K. Alqahtani, Eslam S.A. Bendary, Mahmoud M. Ghuniem, Mohamed A.A. Mahmoud

**Affiliations:** a Food and Nutrition Science Department, Agricultural Science and Food, King Faisal University, Al-Ahsa 31982, Saudi Arabia; b Central Laboratories, King Faisal University, Al-Ahsa 31982, Saudi Arabia; c Department of Agricultural Biochemistry, Faculty of Agriculture, Ain Shams University, P.O. Box 68, Hadayek Shobra, Cairo 11241, Egypt; d Ministry of Agriculture and Land Reclamation, Agricultural Research Center (ARC), Central Laboratory of Residue Analysis of Pesticides and Heavy Metals in Food (QCAP), 7 Nadi Elsaid St, Dokki, Giza 12311, Egypt

**Keywords:** Date palm by-products, Antioxidant phenolics, Nutritional fortification, Lipid oxidation stability, Clean-label bakery

## Abstract

Date palm processing yields underutilized by-products—fiber-rich date press cake (DPC) and lipid-rich date seed oil (DPSO)—with potential as multifunctional, sustainable ingredients. This study assessed their incorporation into cakes (DPC: 12.5–50 %; DPSO: 25–100 %) to improve nutritional, antioxidant, and shelf-life properties. Cakes with DPC had high phenolic and flavonoid contents (TP ≈ 548 mg GAE/100 g, TF ≈ 87 mg QE/100 g), with LC–MS confirming retention of catechin, chlorogenic acid, and quercetin post-baking. These contributed to 94.6 % DPPH radical scavenging, outperforming BHT. DPSO, rich in oleic acid (115.22 μg/g), reduced malondialdehyde by 94 %. Notably, both DPC and DPSO enhanced shelf-life: microbial counts after 21 days were 30–35 % lower than control. DPC-fortified cakes exceeded RDAs for Fe, Mn, and Zn, while remaining below ULs, confirming safety. Sensory evaluation supported acceptability at 12.5 % DPC and all DPSO levels. These findings demonstrate the thermal stability and dual functionality of DPC and DPSO in clean-label bakery applications.

## Highlights

• Date press cake and seed oil were used to fortify cakes at various levels.
• DPC enhanced fiber, minerals, and phenolics; DPSO improved oxidative stability.
• LC–MS confirmed post-baking retention of key flavonoids like catechin and quercetin.
• DPC and DPSO reduced MDA and microbial counts, extending shelf-life naturally.
• Chemometric analysis (PCA) validated the functional and sensory performance patterns.

## Graphical abstract

**Figure:**
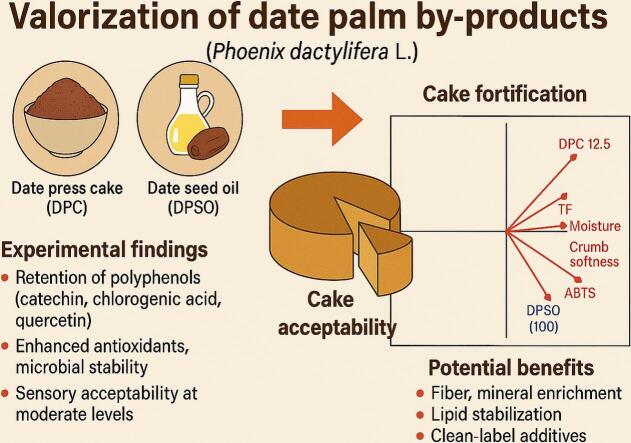

## 1 Introduction

The date palm (*Phoenix dactylifera* L.) is a central crop in arid and semi-arid regions, yielding over 10 million tons of fruit annually (FAOSTAT, 2023). Industrial processing of dates into syrups, pastes, and juices generates substantial volumes of by-products, chiefly date press cake (DPC) and date seed oil (DPSO). Although DPC is rich in dietary fiber, phenolics, and minerals, and DPSO contains unsaturated fatty acids and lipid-soluble antioxidants, these materials remain undervalued in food applications (Alkhalidy et al., 2023; Nehdi et al., 2018).

Industrial processing of dates into syrups, pastes, and juices generates substantial volumes of by-products, chiefly date press cake (DPC) and date seed oil (DPSO). Although DPC is rich in dietary fiber, phenolics, and minerals, and DPSO contains unsaturated fatty acids and lipid-soluble antioxidants, these materials remain undervalued in food applications (Al-Khalili et al., 2023; Karambakhsh et al., 2024). However, major gaps persist. Although previous studies have reported phenolic compounds such as catechin, chlorogenic acid, and quercetin from date fruit and its derivatives, and confirmed their antioxidant activity both in vitro and in vivo (Al-Farsi et al., 2005; Zihad et al., 2021), the thermal stability of these compounds during baking has not been rigorously evaluated. In addition, few studies have addressed whether these antioxidants retain functionality in a baked matrix, or assessed their combined effects on microbiological shelf-life (Karambakhsh et al., 2024; Najjar et al., 2022; Ranasinghe et al., 2022). Similarly, the contribution of date by-products to mineral intake has not been quantified in relation to dietary safety, despite the high levels of transition metals such as Fe and Mn in date press cake.

These issues are critical when considering scale-up for functional bakery products. Without evidence of bioactive retention, oxidative and microbial control, and safe mineral exposure, the practical application of DPC and DPSO in clean-label systems remains uncertain. Prior studies outlined the compositional value of date-derived ingredients, but did not explore these essential dimensions (Al-Khalili et al., 2023; Najjar et al., 2022; Ranasinghe et al., 2022). Even technically focused trials, such as Karambakhsh et al. (2024), omitted evaluation of post-baking antioxidant persistence and dietary intake implications.

To address these gaps, the present study investigates how DPC and DPSO affect the nutritional quality, shelf stability, and sensory properties of fortified cakes. The work includes a detailed analysis of proximate composition, LC–MS profiling of phenolic compounds before and after baking, GC–MS assessment of fatty acid content, antioxidant capacity, microbiological shelf-life over 21 days, and sensory acceptance. Safety was examined using Estimated Provisional Tolerable Daily Intake (EPTDI) calculations for key minerals based on realistic consumption scenarios. Additionally, principal component analysis (PCA) was employed to integrate multivariate data and clarify how formulation variables relate to quality, safety, and sensory outcomes. This multidimensional approach offers new insight into the compatibility and functional performance of DPC and DPSO in cake systems, supporting their use in clean-label product development and circular economy strategies.

## 2 Materials and methods

### 2.1 Raw materials

Khalas date press cake (DPC) and date palm seed oil (DPSO) were obtained from the Al-Jazirah Dates Factory (Al-Hofuf, Al-Ahsa, Saudi Arabia). The DPC was dried at 48 °C in an electric tray dryer for 48 h, then ground to pass through a 0.25-mm sieve (60 mesh) and stored in a glass jar until further use.

### 2.2 Cake ingredients

The cake ingredients included sucrose (commercial grade), palm oil, fresh whole eggs, baking powder (a mixture of sodium bicarbonate and cream of tartar), dry milk powder, and pure vanilla, all of which were purchased from local markets in Al-Hofuf, Saudi Arabia. Soft wheat flour with 72 % extraction rate was also sourced from the local market.

### 2.3 Preparation of cake samples

The cake samples were prepared using a modified method of Ahmed (2014), with formulations incorporating different substitution levels of DPC and DPSO. For the control cake, sugar and palm oil were creamed together for 3 min, followed by the addition of whole eggs, which were mixed for 2 min. Sifted flour, baking powder, and dry milk powder were then incorporated, and the batter was mixed for 4 min. After scraping down the sides of the mixing bowl, an additional 1 min of mixing was performed to ensure homogeneity. A total of 200 ppm of butylated hydroxytoluene (BHT) was further added to the positive control.

The substituted cake batters were prepared by replacing sugar with DPC and palm oil with DSO at varying levels, as shown in Supplementary Table 1. The same mixing procedure used for the control was followed for all substituted formulations. Once prepared, the batters were poured into baking pans and baked in a preheated oven at 180 °C for 30 min. After baking, the cakes were removed from the pans, cooled to room temperature, and packed in polyethylene bags. The cakes were stored at 4 °C for 21 days, with samples taken for analysis at intervals, including within one hour of baking and after 7, 14, and 21 days of storage.

### 2.4 Proximate analysis

For each treatment group, three individual cake samples were homogenized to produce a uniform composite sample. Triplicate measurements were then conducted for each analytical test. Standard procedures from the AOAC (2010) were followed to assess dry matter (DM), crude protein (calculated as %N × 6.25), crude fat, ash, total carbohydrates, and reducing sugars. Crude fiber was quantified using an automated Fibertec 8000 system (FOSS, Denmark).

### 2.5 Trace element analysis by ICP-OES

#### 2.5.1 Microwave-assisted digestion

Samples (∼1 g) were digested following Ghuniem et al. (2022). Each was placed in a microwave digestion vessel with 0.5 mL deionized water, 8 mL concentrated HNO₃, and 2 mL H₂O₂. Vessels were sealed and processed in a microwave system (1800 W, 200 °C for 15 min, then held at 200 °C for another 15 min, followed by cooling to <80 °C). After cooling in a water bath (∼30 min), contents were transferred to 25 mL flasks, diluted with deionized water, and stored in polypropylene tubes for ICP-OES. A reagent blank was prepared similarly.

#### 2.5.2 ICP-OES quantification

Elemental concentrations were measured using a Shimadzu 9820 Series ICP-OES (Tokyo, Japan) equipped with an ASX-280 autosampler and argon plasma. Calibration was performedusing multielement standards (Sigma-Aldrich, 1000 mg/L), covering a range of 0–10 mg/L for all tested elements including Ca, K, Mg, Na, Fe, Mn, Zn, Cu, and Cr.

Estimation of Dietary Intake and Risk Assessment.

To assess dietary exposure and safety, the EPTDI of each element was calculated for each cake formulation based on an assumed daily intake of 47 g/day, representing the average cake consumption in Europe (ReportLinker, 2023), and normalized to a standard adult body weight of 70 kg. The EPTDI was computed using the formula:

$$\mathrm{EPTDI}=\frac{F_{C}\times M_{C}}{B_{W}}\times 10^{-3}\left(\mathrm{mg}/\mathrm{kg}.\mathrm{bw}/\mathrm{day}\right)$$

The EPTDI values were compared to safety limits set by the IOM (2002) and JECFA (2024), including the Recommended Dietary Allowance (RDA) and Tolerable Upper Intake Level (UL). Values above the UL were considered potential safety risks, while those between the RDA and UL were seen as beneficial. Each formulation was assessed for both nutritional adequacy and possible overexposure.

### 2.6 Extraction of lipophilic and phenolic fractions

#### 2.6.1 Hexane extraction (lipophilic removal)

To eliminate lipophilic content, 0.5 g of each sample was extracted twice with 5 mL of 100 % n-hexane (Sigma-Aldrich). The pooled hexane fractions were removed under reduced pressure.

#### 2.6.2 Phenolic extraction and sample preparation

Dried samples (3 g) were extracted with 10 mL of acetone/water/acetic acid (70:29.5:0.5, *v*/v/v) at 25 °C for 2 h with continuous shaking. The mixture was centrifuged (15,000 ×*g*, 20 min, 20 °C), and the extraction was repeated. Supernatants were pooled, evaporated under vacuum, and reconstituted in 100 % methanol to a final volume of 10 mL.

Phenolics from DPSO were extracted using the protocol of Farrés-Cebrián et al. (2016). Oil was mixed with 70 % ethanol (1:1), vortexed, centrifuged, and stored at −18 °C. A hexane wash was used to remove lipids. The ethanolic layer was analyzed via LC–MS using a mobile phase of trichloroacetic acid (0.01 %) and acetonitrile, following a time-resolved gradient over 55 min.

### 2.7 Quantification of total phenolics, tannins, and flavonoids

Total phenolic content was measured using a modified Folin–Ciocalteu method (Goffman & Bergman, 2004). A mixture containing 0.1 mL of extract, 0.5 mL deionized water, 0.25 mL 20 % Folin–Ciocalteu reagent, and 0.5 mL of 0.5 M ethanolamine was incubated for 90 min at room temperature. Absorbance was measured at 760 nm (Thermo Scientific Evolution 350 UV–Vis). Results were expressed as mg gallic acid equivalents (GAE)/100 g dry weight (DW).

Total flavonoids were quantified based on absorbance at 417 nm following reaction with 2 % AlCl₃ (Quettier-Deleu et al., 2000), expressed as mg quercetin equivalents (QE)/100 g DW.

### 2.8 Antioxidant activity

#### 2.8.1 DPPH radical scavenging activity

The DPPH assay was performed following Goffman and Bergman (2004) with modifications. Extracts were mixed with DPPH solution (80 mg/L in methanol), incubated for 30 min at room temperature in the dark, and absorbance was read at 517 nm.

#### 2.8.2 ABTS radical cation assay

The ABTS+ radical cation was generated by mixing 7 mM ABTS with 2.45 mM potassium persulfate and incubating in the dark (12–16 h). The resulting solution was diluted with ethanol. After mixing 0.1 mL of the sample with 0.9 mL ABTS^+^ solution, absorbance at 734 nm was recorded after 1 min.

### 2.9 UPLC–MS identification of phenolic compounds

Phenolic compounds were profiled using a Waters Acquity UPLC–I Class system coupled to a Xevo TQD triple quadrupole mass spectrometer (Waters, Milford, MA, USA) equipped with an electrospray ionization (ESI) source operated in negative ion mode. Separation was achieved on an Acquity UPLC BEH C18 column (2.1 × 100 mm, 1.7 μm) at 25 °C, with a binary gradient of 0.5 % formic acid in deionized water (A) and methanol (B) at 0.8 mL/min. The gradient program was: 0–15 min, 15 % B; 15–25 min, 25 % B; 25–35 min, 50 % B; 35–50 min, 75 % B; 50–55 min, linear return to 15 % B; 55–57 min, isocratic 100 % B for re-equilibration. The injection volume was 20 μL. MS parameters were set as follows: capillary voltage 3000 V, nebulizing gas flow 12 L/h, drying gas temperature 300 °C, nitrogen pressure 60 psi, argon collision gas at 7 psi. Data were acquired in scan mode across *m*/*z* 100–1000. Compound identification was untargeted, performed by comparison with a high-resolution mass spectral library (MassLynx v4.1, Waters) using accurate mass, retention time, MS/MS fragmentation, and isotopic pattern (Elkatry et al., 2023).

### 2.10 GC–MS analysis of fatty acids in DPSO

Fatty acid methyl esters (FAMEs) of DPSO were analyzed using a Shimadzu QP-2010S Plus GC–MS system (Tokyo, Japan) equipped with an AOC-20i + s autosampler and an Rtx-1 capillary column (30 m × 0.32 mm, 0.25 μm). One microliter of the FAME solution was injected in split mode. The oven program was as follows: initial temperature 80 °C, ramped at 10 °C/min to 200 °C (held 5 min), then increased at 15 °C/min to 250 °C and held for 5 min. Injector and interface temperatures were set at 280 °C. Helium was used as the carrier gas at a flow rate of 2.62 mL/min (linear velocity 58.7 cm/s). The ion source temperature was 210 °C. Mass spectra were acquired in EI mode (70 eV) under scan mode from m/z 55–550, with a solvent cut-off time of 4.0 min and acquisition ending at 26.33 min. Peak integration was performed using LabSolutions software v4.1 (Shimadzu, Tokyo, Japan), and compounds were identified by comparison with authentic FAME standards.

### 2.11 Product quality parameters

#### 2.11.1 Weight, volume, and specific volume

The weight and volume of the cake samples were measured one hour after baking to assess their physical properties. Cake volume was determined using the rapeseed displacement method, as described by Randez-Gil et al. (1995), and the specific volume was calculated as the ratio of volume to weight. Additionally, bulk density was calculated as the ratio of the cake's weight (g) to its volume (cm^3^). All measurements were performed in triplicate to ensure accuracy and consistency.

#### 2.11.2 Color analysis

The crust and crumb color of the cake samples were determined using a Minolta Colorimeter (CR 200, Japan) based on the Hunter L*, a*, and b* color space values. Measurements followed the tristimulus color system described by Francis (1983).

### 2.12 Shelf-life assessment

#### 2.12.1 Lipid peroxidation

Malondialdehyde (MDA) was quantified using a commercial TBARS kit (MyBioSource, MBS480427) as described by Papastergiadis et al. (2012). Five grams of dry sample were homogenized with 15 mL of deionized water, then reacted with TBA reagent for 1 h at room temperature. After centrifugation (15,000 ×*g*, 5 min, 20 °C), the supernatant absorbance was measured at 532 nm. MDA concentrations were calculated against a standard curve (0.047–3.0 mg/L).

#### 2.12.2 Microbiological analysis

The microbiological analysis was conducted according to the method of Łopusiewicz (2023), with slight modifications. A 10 g sample of cake (crumb and crust) was taken and placed into a sterile stomacher bag under aseptic conditions. The samples were diluted with 90 mL of sterile physiological saline (0.9 %) and homogenized for one minute using a Bag Mixer (Interscience, Saint-Nom-la-Brèteche, France).

Appropriate decimal dilutions were prepared in sterile buffered peptone water (Merck, Darmstadt, Germany). Total microbial counts were enumerated on Plate Count Agar (Merck, Darmstadt, Germany) and incubated at 37 °C under aerobic conditions for 24 h. For molds and yeasts, 1 mL of the diluted sample was spread-plated on potato dextrose agar and incubated at 28 °C for 72 h under aerobic conditions. The viable cell counts were expressed as log CFU/g of the samples. Microbial enumeration was performed in triplicate by counting plates with 30–300 colonies.

### 2.13 Sensory evaluation

Sensory evaluation was conducted by a panel of ten trained members (5 males and 5 females, aged 30–55 years) from the Department of Food Science and Nutrition, King Faisal University. Panelists assessed the cake samples for appearance, crust color, crumb color, texture, taste, odor, and overall acceptability using a 10-point hedonic scale, following the protocol outlined by AACC (2000), where 1 represented “dislike extremely” and 10 indicated “like extremely.” All samples were coded and presented in randomized order under standardized lighting and temperature conditions. The study was approved by the King Faisal University Research Ethics Committee under approval number KFU-2025-Ethics-3445.

### 2.14 Statistical analysis

Principal component analysis (PCA) was performed using XLSTAT software (version 2024.1, Addinsoft, Paris, France). Statistical analysis was conducted using one-way ANOVA to identify significant differences among samples. Tukey's HSD test was used for pairwise comparisons, and groups sharing the same letter were not significantly different (*p* < 0.05). Normality and homogeneity of variance were checked before analysis. All other statistical computations and visualizations were carried out in Python using scipy, statsmodels, and pandas, with plots created using matplotlib and seaborn.

## 3 Results and discussion

### 3.1 Proximate composition

#### 3.1.1 Composition of raw materials

The two fortifying agents (DPC and DPSO) differed markedly in their proximate and lipid composition, reflecting their distinct origins and processing methods (Table 1).

**Table 1:** Proximate composition of raw materials and fortified cake samples (%, dry matter basis), and fatty acid profile of date seed oil (μg/g).

Sample	Proximate Composition						
	Dry Matter %	Ash%	Crude Fiber%	Crude Fat%	Crude Protein%	Total Carbohydrates%	Total Reducing Sugars%
DPC	95.38 ± 0.03ᵇ	2.76 ± 0.05ᵃ	14.35 ± 0.05ᵃ	4.00 ± 0.10ᵃ	10.55 ± 0.05ᵈ	61.65 ± 0.35ᵃ	25.90 ± 0.10ᵃ
Control	98.12 ± 0.34ᵃ	0.56 ± 0.01ᶠ	0.99 ± 0.01ᵉ	19.67 ± 0.05ᵉ	7.68 ± 0.09ᶜ	45.58 ± 0.14ᵇ	20.58 ± 0.02ᵇ
DPC-12.5	94.74 ± 0.24ᶜ	0.60 ± 0.01ᵈᵉ	1.18 ± 0.00ᵈ	19.79 ± 0.04ᵈ	7.88 ± 0.02ᶜ	44.10 ± 0.13ᶜ	18.96 ± 0.00ᶜ
DPC-25	94.32 ± 0.42ᶜ	0.67 ± 0.01ᶜ	1.56 ± 0.00ᶜ	19.96 ± 0.02ᶜ	8.33 ± 0.17ᵇ	42.80 ± 0.17ᵈ	17.73 ± 0.01ᵈ
DPC-50	94.64 ± 0.14ᶜ	0.78 ± 0.01ᵇ	2.57 ± 0.03ᵇ	20.26 ± 0.13ᵇ	8.88 ± 0.13ᵃ	42.46 ± 0.04ᵉ	14.04 ± 0.01ᵉ
DPSO-25	95.80 ± 0.14ᵇ	0.57 ± 0.01ᵉᶠ	0.93 ± 0.03ᶠ	7.64 ± 0.04ᵉ	7.67 ± 0.02ᶜ	45.51 ± 0.03ᵇ	20.59 ± 0.01ᵇ
DPSO-50	95.68 ± 0.38ᵇ	0.59 ± 0.01ᵈᵉᶠ	0.98 ± 0.02ᵉ	7.67 ± 0.04ᵉ	7.77 ± 0.02ᶜ	45.64 ± 0.20ᵇ	20.60 ± 0.01ᵇ
DPSO-100	98.02 ± 0.24ᵃ	0.62 ± 0.01ᵈ	0.95 ± 0.01ᵉᶠ	7.64 ± 0.04ᵉ	7.77 ± 0.02ᶜ	45.58 ± 0.14ᵇ	20.58 ± 0.01ᵇ
	Fatty Acids Composition						
	Lauric acid (C12:0)	Palmitic acid (C16:0)	Stearic acid (C18:0)	Palmitoleic acid C16:1 (cis-9)	Oleic acid (C18:1 cis-9)	Linolenic acid (C18:2)	Docosadienoic acid (C22:2)
	6.41	52.99	28.42	8.70	115.22	269.47	9.61
RT (min)	10.8	11.9	12.3	12.6	12.9	13.3	14.1
DPSO	SFAs			MUFAs		PUFAs	
	17.89 %		25.25 %			56.86 %	

Values are means ± SD (*n* = 3). Within each column, different superscript letters indicate significant differences (*p* < 0.05). Fatty acid composition refers to the profile of date seed oil (DPSO) and includes individual fatty acids as well as grouped totals for saturated (SFAs), monounsaturated (MUFAs), and polyunsaturated fatty acids (PUFAs).

Date press cake was high in total carbohydrates (43.3 %), of which a substantial portion was crude fiber (36.8 %), consistent with the fibrous nature of the material following juice and oil extraction. It also contained moderate levels of protein (10.4 %), ash (4.8 %), and a low level of fat (1.2 %). These results align with previous studies reporting that DPC, derived from the fibrous residues of date fruit and pits, is rich in insoluble dietary fiber and minerals but contributes little to fat content (Al-Farsi et al., 2005; Noorbakhsh, 2023).

Date seed oil contained 56.86 % polyunsaturated fatty acids (PUFAs), with linoleic acid (C18:2) as the dominant component (269.47 μg/g), followed by oleic acid (C18:1; 115.22 μg/g) and smaller contributions from saturated fatty acids like palmitic acid (C16:0; 52.99 μg/g) and stearic acid (C18:0; 28.42 μg/g). Notably, linolenic acid (C18:3) was detected at a substantially higher level than in previous reports, contributing to the observed 56.86 % PUFA fraction. Earlier studies typically described DPSO as predominantly monounsaturated, with oleic acid around 45–55 %, linoleic acid near 10–15 %, and negligible linolenic acid (<1 %) (Al-Farsi et al., 2005; Nehdi et al., 2018). This compositional shift may reflect varietal differences, oil extraction conditions, or postharvest handling. The elevated linolenic acid content is nutritionally favorable due to its omega-3 properties but introduces higher oxidative risk.

#### 3.1.2 Effect on fortified cake composition

Incorporation of DPC and DPSO into cake formulations led to notable shifts in proximate composition compared to the control. Ash content increased significantly with DPC inclusion, reaching 2.76 % in the raw DPC and rising from 0.60 % to 0.78 % across the 12.5 % to 50 % DPC formulations, versus only 0.56 % in the control. This reflects the mineral richness of DPC, particularly in potassium, calcium, and iron, consistent with previous reports (Al-Farsi et al., 2005; Mahomoodally et al., 2024). DPSO, being a refined oil, had negligible impact on ash content.

Crude fiber increased progressively with higher DPC levels (1.18 % to 2.57 %), confirming DPC's role as a dietary fiber enhancer. The raw DPC contained 14.35 % fiber, supporting its classification as a high-fiber by-product suitable for clean-label fortification. Fiber enrichment not only adds nutritional value but also contributes to improved satiety and potential glycemic moderation (Das et al., 2023). In contrast, fiber content in DPSO and control cakes remained below 1 %, highlighting DPC as the principal fiber source.

Fat content decreased with DPC addition due to its defatted nature, dropping from 19.67 % in the control to 20.26 %, 19.96 %, and 19.79 % in DPC-fortified cakes, while raw DPC itself contained only 4.00 %. This dilution effect could benefit low-fat formulation targets. DPSO-fortified cakes retained fat levels between 7.64 % and 7.67 %, confirming DPSO's role in lipid phase continuity, which is relevant for texture and moisture retention (Hamza et al., 2021; Nehdi et al., 2018).

Protein content was slightly elevated in DPC-fortified samples, peaking at 8.88 % in the 50 % DPC formulation versus 7.68 % in the control. This increase may result from the relative concentration of proteins as fat and carbohydrate components are displaced. Protein levels in DPSO cakes remained similar to the control (∼7.67–7.77 %).

Available carbohydrates decreased with DPC inclusion (from 45.58 % in the control to 42.46 % at DPC-50), likely due to starch displacement by non-digestible fiber. This is nutritionally desirable in reducing glycemic impact, as supported by Ouamnina et al. (2024). Meanwhile, DPSO cakes maintained stable carbohydrate levels, as flour and sugar content were not altered.

Reducing sugars peaked in the raw DPC (25.90 %) but declined with DPC incorporation into the cake (18.96 % to 14.04 %), showing a dilution trend. This could also indicate participation of sugars in Maillard reactions during baking, contributing to browning and flavor development (Prakash et al., 2022). DPSO cakes retained reducing sugar levels around 20.58–20.60 %, comparable to the control.

### 3.2 Mineral content and nutritional safety assessment

The mineral composition of the cake samples was significantly influenced by the addition of date press cake, with milder changes observed in those fortified with date seed oil. Raw DPC exhibited high concentrations of potassium (190.35 mg/kg), iron (15.30 mg/kg), and manganese (10.43 mg/kg), consistent with previous reports highlighting the mineral richness of date-based by-products (Mahomoodally et al., 2024; Muñoz-Tebar et al., 2024). In contrast, raw DPSO contained only trace minerals, reflecting losses during oil extraction (Nehdi et al., 2018).

Incorporation of DPC into cakes resulted in a progressive increase in several key minerals. Potassium rose from 17.67 mg/kg in the control to 39.61 mg/kg in DPC-50 (*p* < 0.05), while calcium increased from 15.99 mg/kg to 19.33 mg/kg. Iron and manganese also followed this trend, reaching 2.52 mg/kg and 1.58 mg/kg in DPC-50, respectively, compared to 1.00 mg/kg and 0.47 mg/kg in the control. These increases align with the natural mineral profile of date seed fiber (Al-Farsi et al., 2005; Mahomoodally et al., 2024).

Trace elements such as copper and chromium, undetectable in both control and DPSO-fortified cakes, were only present in the DPC groups—further confirming their origin from the fibrous seed matrix. Zinc levels showed a modest increase with DPC, rising to 0.71 mg/kg in DPC-50 compared to 0.59 mg/kg in the control, though this difference was not statistically significant.

Interestingly, magnesium content declined across DPC and DPSO formulations. The control sample had the highest value (125.97 mg/kg), which decreased to 111.23 mg/kg in DPC-50 and 104.14 mg/kg in DPSO-100. This reduction may be attributed to thermal instability or interactions within the cake matrix that affect magnesium retention (Mahomoodally et al., 2024).

Overall, DPSO contributed minimally to the mineral profile. Levels of potassium, calcium, iron, and chromium remained low across all DPSO cakes, consistent with the limited mineral content of the oil phase and confirming the nutritional enrichment effect of DPC alone.

Estimated Provisional Tolerable Daily Intake (EPTDI) values were calculated for key elements in cakes fortified with date-derived additives, based on a daily intake of 47 g and a 70 kg body weight. This calculation is essential for evaluating whether the intake of minerals such as iron, zinc, and manganese remains within safe and beneficial limits when consumed regularly. By estimating the actual exposure through fortified foods, EPTDI provides a realistic framework to assess both nutritional adequacy and toxicological safety. Results (Table 2) were evaluated against RDA and UL thresholds from IOM (2002) and JECFA (2024) to assess nutritional adequacy and safety.

**Table 2:** Mineral Concentration (mg/kg DW) and Estimated Provisional Tolerable Daily Intake (EPTDI, mg/kg bw/day) in Cakes Containing Date-Based Additives.

	Concentration per mg/Kg^a^								
Sample	Na	K	Ca	Mg	Fe	Mn	Zn	Cu	Cr
DPC	16.59 ± 0.04^h^	190.35 ± 10.05^a^	42.10 ± 0.60^a^	20.18 ± 0.08^g^	15.30 ± 0.30^a^	10.43 ± 0.05^a^	2.64 ± 0.16^a^	0.28 ± 0.02^a^	0.08 ± 0.00^a^
DPSP	1.34 ± 0.00^i^	0.00 ± 0.00^e^	0.02 ± 0.02^h^	0.00 ± 0.00^h^	0.00 ± 0.00^f^	1.54 ± 0.01^c^	0.01 ± 0.00^g^	0.00 ± 0.00^d^	0.00 ± 0.00^e^
Control	26.69 ± 0.03^d^	17.67 ± 0.93^d^	15.99 ± 0.02^e^	125.97 ± 0.47^a^	1.00 ± 0.00^e^	0.47 ± 0.00^i^	0.59 ± 0.03^cd^	0.00 ± 0.00^d^	0.00 ± 0.00^e^
DPC-12.5	22.88 ± 0.05^g^	25.93 ± 1.37^c^	16.98 ± 0.12^d^	120.65 ± 0.03^b^	1.38 ± 0.01^d^	0.77 ± 0.00^e^	0.56 ± 0.00^cde^	0.008 ± 0.00^cd^	0.003 ± 0.00^d^
DPC-25	27.79 ± 0.08^c^	28.78 ± 1.52^c^	17.70 ± 0.11^c^	118.24 ± 0.50^c^	1.73 ± 0.04^c^	1.03 ± 0.01^d^	0.64 ± 0.04^bc^	0.014 ± 0.00^bc^	0.005 ± 0.00^c^
DPC-50	30.93 ± 0.08^a^	39.61 ± 2.09^b^	19.33 ± 0.14^b^	111.23 ± 1.36^e^	2.52 ± 0.01^b^	1.58 ± 0.00^b^	0.71 ± 0.05^b^	0.024 ± 0.00^b^	0.007 ± 0.00^b^
DPSO-25	30.43 ± 0.07^b^	17.65 ± 0.95^d^	15.95 ± 0.10^e^	117.46 ± 0.46^c^	0.97 ± 0.00^e^	0.55 ± 0.00^h^	0.50 ± 0.00^def^	0.00 ± 0.00^d^	0.00 ± 0.00^e^
DPSO-50	25.68 ± 0.06^e^	13.49 ± 0.71^d^	15.20 ± 0.10^f^	112.66 ± 0.42^d^	0.92 ± 0.00^e^	0.58 ± 0.00^g^	0.47 ± 0.00^ef^	0.00 ± 0.00^d^	0.00 ± 0.00^e^
DPSO-100	24.98 ± 0.06^f^	15.96 ± 0.84^d^	14.01 ± 0.09^g^	104.14 ± 0.39^f^	0.85 ± 0.00^e^	0.67 ± 0.00^f^	0.42 ± 0.00^f^	0.00 ± 0.00^d^	0.00 ± 0.00^e^
	EPTDI (mg/kg bw/day) in Fortified Cake Samples								
	Na	K	Ca	Mg	Fe	Mn	Zn	Cu	Cr
DPC-12.5	1.54 × 10^−2^	1.74 × 10^−2^	1.14 × 10^−2^	8.10 × 10^−2^	9.26 × 10^−4^	5.17 × 10^−4^	3.76 × 10^−4^	5.06 × 10^−6^	1.68 × 10^−6^
DPC-25	1.87 × 10^−2^	1.93 × 10^−2^	1.19 × 10^−2^	7.94 × 10^−2^	1.16 × 10^−3^	6.91 × 10^−4^	4.32 × 10^−4^	9.42 × 10^−6^	3.35 × 10^−6^
DPC-50	2.08 × 10^−2^	2.66 × 10^−2^	1.30 × 10^−2^	7.47 × 10^−2^	1.69 × 10^−3^	1.06 × 10^−3^	4.79 × 10^−4^	1.63 × 10^−5^	4.48 × 10^−6^
DPSO-25	2.04 × 10^−2^	1.19 × 10^−2^	1.07 × 10^−2^	7.89 × 10^−2^	6.53 × 10^−4^	3.66 × 10^−4^	3.37 × 10^−4^	0	0
DPSO-50	1.72 × 10^−2^	9.06 × 10^−3^	1.02 × 10^−2^	7.56 × 10^−2^	6.19 × 10^−4^	3.91 × 10^−4^	3.18 × 10^−4^	0	0
DPSO-100	1.68 × 10^−2^	1.07 × 10^−2^	9.41 × 10^−3^	6.99 × 10^−2^	5.73 × 10^−4^	4.47 × 10^−4^	2.79 × 10^−4^	6.75 × 10^−7^	0

a Values are means ± SD (*n* = 3). Within each column, different superscript letters indicate significant differences (*p* < 0.05).

Among all fortified samples, cakes containing DPC exhibited markedly higher EPTDI values for most elements compared to those fortified with DPSO. This trend reflects the inherently richer mineral composition of DPC, as previously reported by Ranasinghe et al. (2022). The DPC-50 formulation, in particular, yielded the highest EPTDI values for Fe (1.69 × 10^−3^ mg/kg bw/day), Mn (1.06 × 10^−3^ mg/kg bw/day), and Zn (4.79 × 10^−4^ mg/kg bw/day), suggesting its superior capacity to contribute to micronutrient intake.

Importantly, all calculated EPTDI values across formulations remained well below the UL limits, indicating that none of the tested fortification levels pose a risk of mineral overconsumption. For key micronutrients such as iron, manganese, and zinc, the EPTDI values in the DPC-25 and DPC-50 samples surpassed the RDA but remained safely within UL thresholds. This pattern suggests that DPC fortification contributes meaningfully to daily mineral intake while maintaining a wide toxicological safety margin, supporting its use as a nutritionally beneficial and regulatorily safe ingredient in functional bakery applications.

The contribution of DPSO to mineral intake was limited. EPTDI values for DPSO-fortified cakes were substantially lower for all minerals assessed, which is consistent with the refining process used in oil extraction and its inherently lower ash and mineral content. For instance, Cu and Cr were undetectable in DPSO-25 and 50 samples, while DPSO-100 exhibited only trace levels (e.g., 6.75 × 10^−7^ mg/kg bw/day for Cu), far below any biological significance.

From a nutritional standpoint, DPC fortification—particularly at higher levels—offers a practical approach to addressing iron and zinc deficiencies through bakery products, without exceeding safety limits. Its mineral content supports clean-label and functional food goals. In contrast, DPSO contributes little to mineral intake, highlighting the need for complementary ingredients if broader nutritional benefits are desired.

### 3.3 Total phenolic and flavonoid content

The total phenolic (TP) and flavonoid (TF) content of the raw date press cake was found to be remarkably high, reaching 1340.3 ± 16.4 mg GAE/100 g and 1204.7 ± 4.7 mg QE/100 g, respectively (Table 3). These values underscore DPC's potential as a rich source of polyphenols for food fortification (Al-Farsi et al., 2005; Das et al., 2023; Zihad et al., 2021). In contrast, TP and TF were not measured in the raw date seed oil, due to its minimal phenolic solubility in aqueous extraction media.

**Table 3:** Phenolic composition of oil and press cake determined by LC–MS.

Compound	RT (min)	m/z [M–H]^−^	DPSO	DPC	DPC-12.5	DPC-25	DPC-50	
Gallic acid	0.37	171.1	ND	597.74	214.20	230.47	299.89	
Chlorogenic acid	10.31	355.1	ND	781.15	202.70	155.28	283.78	
Syringic acid	11.56	199.1	ND	507.22	102.55	110.43	143.57	
Vanillic acid	11.90	169	ND	885.02	ND	ND	63.94	
Catechin	12.14	291.1	356.00	2129.09	158.65	192.47	222.11	
Epicatechin	12.56	291.1	ND	260.86	339.23	408.35	474.93	
Caffeic acid	13.08	181	ND	339.56	ND	ND	ND	
Luteolin-7-*O*-glucoside	14.06	449.1	ND	1598.23	ND	ND	ND	
Rutin	16.32	611.2	ND	429.96	97.13	288.69	330.23	
Luteolin	17.28	287	167.10	1550.32	ND	ND	ND	
Vitexin	17.84	313.1	ND	199.32	68.11	84.54	95.35	
Apigenin	17.95	271	ND	419.10	ND	ND	ND	
Quercetin	18.20	303	ND	1658.15	ND	ND	ND	
Naringenin	19.51	273	ND	236.89	ND	ND	ND	
Kaempferol	19.89	287	ND	336.66	ND	ND	ND	
Ellagic acid	22.83	303	ND	403.87	ND	ND	ND	
Protocatechuic acid	6.20	155	ND	784.38	ND	ND	ND	
Procyanidin	60.98	867	ND	371.42	ND	ND	ND	
Total phenolic (mg GAE/100 g DW) Flavonoids (mg QE/100 g DW)								
	DPC	Control	DPC-12.5	DPC-25	DPC-50	DPSO-25	DPSO-50	DPSO-100
TP	1340.30 ± 16.35ᵃ	7.06 ± 0.09ᶠ	75.98 ± 0.93ᵈ	262.45 ± 3.20ᶜ	548.31 ± 6.69ᵇ	21.94 ± 0.27ᵉ	20.69 ± 0.25ᵉ	15.49 ± 0.19ᵉᶠ
TF	1204.68 ± 4.68ᵃ	0.00 ± 0.00ᵉ	10.04 ± 0.04ᵈ	26.77 ± 0.10ᶜ	87.00 ± 0.34ᵇ	0.00 ± 0.00ᵉ	0.17 ± 0.001ᵉ	0.17 ± 0.001ᵉ

**ND** = Not Detected. Values are means ± SD (*n* = 3). Within each column, different superscript letters indicate significant differences (*p* < 0.05).

When incorporated into cake formulations, DPC significantly enhanced TP and TF content in a concentration-dependent manner (*p* < 0.05). Cakes fortified with 12.5 %, 25 %, and 50 % DPC yielded TP values of 75.98 ± 0.93, 262.45 ± 3.20, and 548.31 ± 6.69 mg GAE/100 g, respectively. Corresponding TF values were 10.04 ± 0.04, 26.77 ± 0.10, and 87.00 ± 0.34 mg QE/100 g. These increases reflect the partial retention of heat-stable polyphenols from the DPC matrix after baking, consistent with previous studies on fiber-bound phenolics in thermally processed products (Prakash et al., 2022).

In comparison, cakes fortified with DPSO exhibited very low TP levels (15.49–21.94 mg GAE/100 g) and negligible TF (≤0.17 mg QE/100 g), with no significant difference from the unfortified control. This confirms that most hydrophilic phenolics remain in the press cake after oil extraction and that DPSO contributes little to phenolic enrichment when assessed by standard solvent-based assays (Ouamnina et al., 2024).

Notably, the TP measured in the 12.5 % DPC cake (75.98 ± 0.93 mg GAE/100 g) was lower than the proportional expectation (∼167.5 mg/100 g based on raw DPC), corresponding to ∼45 % recovery versus ∼78–82 % at 25–50 % DPC. This non-linearity reflects concentration-dependent matrix and process effects rather than analytical error. A substantial fraction of DPC phenolics are fiber-bound/non-extractable, and at low inclusion levels these forms are more strongly sequestered within the starch–protein–sugar matrix, reducing solvent extractability and Folin response. In addition, thermal/Maillard interactions (phenolic–protein/sugar conjugation) further decrease solubility and reagent reactivity, disproportionately affecting low-concentration phenolics (Das et al., 2023; Prakash et al., 2022). As DPC increases, the phenolic pool exceeds matrix binding capacity, improving recovery and producing the sharp rise at 25–50 %. The same rationale explains the weaker Concentration-dependence observed for TF, as flavonoids are more labile and prone to complexation. Overall, these findings suggest that phenolic retention in baked products is not strictly linear with raw ingredient content but is strongly influenced by food matrix interactions and thermal processing.

Phenolic Compound Profiling by LC–MS.

Consistent with the non-linear recovery trends observed in Folin-based TP and TF assays, untargeted LC–MS/MS profiling was performed to validate the actual phenolic spectrum and retention in DPC- and DPSO-fortified cakes. The untargeted LC–MS/MS profiling confirmed a much broader and richer phenolic spectrum in DPC-fortified cakes compared to those containing only DPSO (Table 3). Using a high-resolution mass spectral library for compound confirmation, a total of 18 phenolics were identified in the DPC extract, spanning flavan-3-ols, flavonols, flavones (both aglycone and glycoside forms), phenolic acids, and hydrolyzable tannins. By contrast, DPSO contained only two phenolics—catechin (356.0 μg/g) and luteolin (167.1 μg/g)—both detected at relatively low levels, underscoring its limited contribution to polyphenol enrichment.

Among the phenolics detected in DPC, catechin (2129.1 μg/g), quercetin (1658.2 μg/g), luteolin (1550.3 μg/g), and luteolin-7-O-glucoside (1598.2 μg/g) were predominant. Substantial levels of phenolic acids were also observed, including vanillic acid (885.0 μg/g), protocatechuic acid (784.4 μg/g), and chlorogenic acid (781.2 μg/g). Additional compounds such as rutin (∼430 μg/g), vitexin (∼199 μg/g), ellagic acid (∼404 μg/g), and procyanidin (∼371 μg/g) were exclusively found in DPC. These findings align with previous reports indicating that hydrophilic and high-molecular-weight phenolics are retained in the fibrous residue after juice or oil extraction (Zihad et al., 2021).

The absence of most phenolics in DPSO is consistent with the poor solubility of these compounds in non-polar solvents. Phenolic acids, flavonoid glycosides, and tannins are more likely to remain in aqueous or fiber-rich matrices, which explains their limited presence in the oil fraction (El Kamari et al., 2024; Hamza et al., 2021). Although prior studies have reported trace amounts of vanillic, ferulic, and protocatechuic acids in DPSO, these were not detected here—likely due to processing losses or low native concentrations (Hamza et al., 2021).

### 3.4 Residual phenolic compounds in baked DPC-fortified cakes

To evaluate the thermal stability of phenolic compounds, LC–MS profiling was conducted on cakes fortified with varying levels of DPC. The results confirmed that key phenolics were retained post-baking in a concentration-dependent manner across the 12.5 %, 25 %, and 50 % DPC formulations.

Gallic acid increased from 214.2 μg/g in the 12.5 % DPC cake to 299.9 μg/g at 50 %. Protocatechuic and chlorogenic acids followed a similar trend, reaching 283.8 and 143.6 μg/g, respectively, at 50 % DPC. Vanillic acid was also present at notable levels (158.7–222.1 μg/g), while syringic acid emerged only in the 50 % DPC sample (63.9 μg/g), indicating a possible threshold effect in extractability or thermal stability.

Among flavonoids, catechin was the most abundant, increasing from 339.2 to 474.9 μg/g. Luteolin and its glucoside were consistently detected, with the latter peaking at 330.2 μg/g in the 50 % DPC cake. These findings align with previous reports indicating the thermal resilience of date-derived polyphenols during baking (Prakash et al., 2022; Ranasinghe et al., 2022).

The persistence of both aglycone and glycosylated phenolics suggests limited thermal degradation and supports DPC's role as an effective carrier of bioactive antioxidants. The retention of catechin, gallic acid, chlorogenic acid, and quercetin derivatives underscores the functional and nutritional potential of DPC-enriched bakery products (Al-Farsi et al., 2005; El Kamari et al., 2024; Zihad et al., 2021).

### 3.5 Antioxidant activity

Antioxidant capacity was evaluated using DPPH and ABTS radical scavenging assays. Vitamin C served as a reference standard. Among the raw materials, only date press cake was directly evaluated. Cake samples fortified with DPC or DPSO were then compared against an unfortified control (negative) and a BHT-supplemented control (positive). Statistical differences were considered significant at *p* < 0.05 (Supplementary Fig. 1).

Raw DPC showed potent antioxidant activity, achieving 94.6 % inhibition in DPPH and 88.7 % in ABTS assays, statistically comparable to vitamin C. This high activity is consistent with its elevated levels of thermally stable polyphenols, particularly catechin, chlorogenic acid, and quercetin, identified via LC–MS. These compounds are known for their redox properties, acting as hydrogen donors and metal chelators (Al-Farsi et al., 2005; Prakash et al., 2022).

In fortified cakes, antioxidant activity scaled with DPC concentration. DPC-50 showed 53.7 % (DPPH) and 41.6 % (ABTS) inhibition, while DPC-25 and DPC-12.5 followed in a concentration-dependent pattern (*p* < 0.05). These results demonstrate that a substantial portion of polyphenols retained functionality post-baking, likely due to their matrix-binding stability and resistance to thermal degradation (Ranasinghe et al., 2022; Zihad et al., 2021).

By contrast, DPSO-fortified cakes exhibited minimal radical scavenging (≤8 % for both assays), with no significant difference from the control (*p* > 0.05). This outcome aligns with DPSO's low content of hydrophilic phenolics and highlights the limitations of DPPH and ABTS assays in detecting lipid-phase antioxidants. Tocopherols, though abundant in DPSO, are poorly soluble in methanol and more relevant in lipid oxidation suppression, as evidenced by TBARS analysis (Al-Khalili et al., 2023; Nehdi et al., 2018).

These findings emphasize the differing modes of action: DPC acts primarily in aqueous systems via polyphenol-driven radical scavenging, while DPSO functions in lipid matrices, stabilizing unsaturated fatty acids and interrupting lipid peroxidation. This division of functionality supports their combined use in bakery systems for comprehensive oxidative protection.

### 3.6 Cake quality parameters

#### 3.6.1 Physical properties

**Fig. 1:**
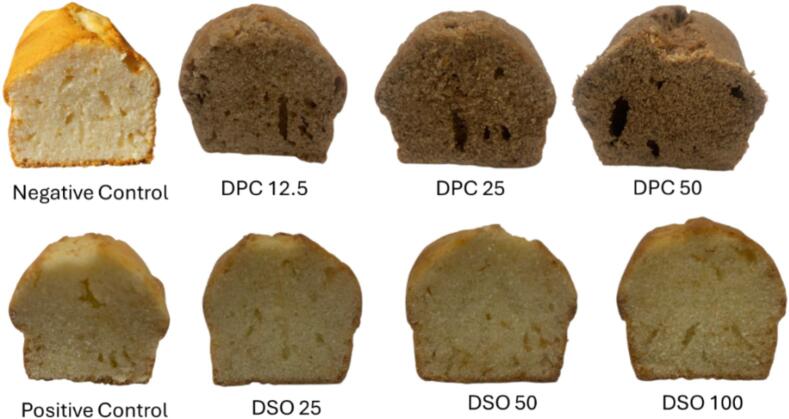
Cross-sectional images of cakes formulated with different levels of date press cake and date seed oil, compared to control samples.

The incorporation of date press cake and date seed oil affected key physical attributes of the cakes, including weight, height, volume, specific volume, and bulk density (Table 4; Fig. 1). DPC fortification, particularly at higher levels (25–50 %), led to denser and flatter cakes, while DPSO substitution maintained structural properties close to the control.

**Table 4:** Physical parameters of for cakes with varying substitution levels of date press cake and date seed oil.

Samples	Weight	Height	Volume	*S*volume	Bulk Density	Height-to-Weight Ratio
Negative Control	402.0 ± 1.0 ^ab^	6.90 ± 0.10 ^ab^	618.33 ± 22.87 ^ab^	1.54 ± 0.06 ^ab^	0.65 ± 0.03 ^bc^	0.017 ± 0.00 ^ab^
Positive Control	404.0 ± 11.14 ^ab^	7.00 ± 0.10 ^a^	634.0 ± 5.29 ^a^	1.57 ± 0.05 ^ab^	0.64 ± 0.02 ^bc^	0.017 ± 0.0 ^ab^
DPC-12.5	405.0 ± 1.0 ^a^	6.73 ± 0.15 ^bc^	653.33 ± 5.77 ^a^	1.61 ± 0.02 ^a^	0.62 ± 0.00 ^c^	0.017 ± 0.00 ^a^
DPC-25	402.0 ± 1.0 ^ab^	6.20 ± 0.10 ^d^	530.0 ± 10.0 ^c^	1.32 ± 0.03 ^c^	0.76 ± 0.01 ^a^	0.015 ± 0.00 ^c^
DPC-50	401.0 ± 1.0 ^ab^	6.10 ± 0.10 ^d^	530.0 ± 20.0 ^c^	1.32 ± 0.06 ^c^	0.76 ± 0.03 ^a^	0.015 ± 0.00 ^c^
DSO-25	403.0 ± 3.0 ^ab^	6.73 ± 0.06 ^bc^	620.0 ± 40.0 ^ab^	1.54 ± 0.10 ^ab^	0.65 ± 0.04 ^bc^	0.017 ± 0.00 ^ab^
DSO-50	399.0 ± 1.0 ^ab^	6.70 ± 0.10 ^bc^	596.67 ± 15.28 ^b^	1.50 ± 0.03 ^b^	0.67 ± 0.02 ^d^	0.017 ± 0.00 ^b^
DSO-100	396.33 ± 1.53 ^b^	6.63 ± 0.15 ^c^	585.0 ± 13.23 ^b^	1.48 ± 0.03 ^b^	0.68 ± 0.01 ^d^	0.0167 ± 0.00 ^b^

Values are expressed as mean ± SD (*n* = 3). Different letters indicate statistically significant differences among treatments within each assay (*p* < 0.05).

Final cake weights were comparable (∼396–405 g) across treatments, with no significant differences (*p* > 0.05), except for a slight reduction in DPSO-100 (*p* < 0.05), possibly due to greater moisture loss (Ahmed et al., 2023; Kaur et al., 2022). Cake height and volume decreased significantly with increasing DPC, with DPC-50 showing the lowest height (∼6.10 cm). In contrast, DPC-12.5 and all DPSO treatments remained comparable to the control (∼6.7–7.0 cm; *p* > 0.05), indicating acceptable aeration at low DPC levels. These reductions in expansion are consistent with reports on DPC sponge cakes, where higher substitution (20–40 %) increased batter consistency and density, reduced air cell retention, and delayed starch gelatinization, ultimately lowering cake height and volume (Karambakhsh et al., 2024).

Specific volume was highest in DPC-12.5 (1.61 mL/g), reflecting a well-aerated structure, while bulk density increased at higher DPC levels. These changes align with previous studies showing that fiber addition increases batter viscosity, reducing expansion (Jahan et al., 2023; Karambakhsh et al., 2024; Yunusa et al., 2025). Visual inspection confirmed that high-DPC cakes had denser crumbs with smaller air cells, whereas DPSO-100 cakes resembled the control (Kaur et al., 2022).

DPSO substitution (up to 100 %) preserved volume and height, with only minor reductions. This suggests that despite being a liquid oil, DPSO can act as a suitable fat replacer due to its compatibility with cake aeration mechanisms, aided by emulsifiers in the formulation (Kaur et al., 2022).

#### 3.6.2 Color attributes

Fortification with DPC and DPSO resulted in significant changes in the color properties of the cakes, measured by L* (lightness), a* (redness), and b* (yellowness) values, as well as total color difference (Δ*E*; Supplementary Fig. 2). DPC addition caused a notable darkening of both crust and crumb, while DPSO had only minimal effects on visual attributes.

In the crumb, increasing DPC levels progressively lowered L* and elevated a*, indicating a shift toward darker, red-brown hues (*p* < 0.05). DPC-50 displayed the most pronounced change, with a Δ*E* of approximately 39.3 compared to the control—well beyond the visual detection threshold. The b* value declined with DPC, further dulling the yellow tone. In contrast, DPSO-fortified samples retained a light crumb; DPSO-100 exhibited the highest b* (∼29.9) and a low Δ*E* (∼3.96), suggesting minimal perceptible difference from the control.

Similar trends were observed in the crust. DPC-50 had the lowest L* (∼35.73) and b* (∼17.78), and the highest a* (∼10.58), yielding a Δ*E* of ∼37.8. Conversely, DPSO-100 maintained higher L* and b* values, close to the control, with only a moderate Δ*E* (∼11.2). These data suggest that DPC incorporation leads to visually darker and redder baked surfaces, whereas DPSO preserves the characteristic golden crust.

The darkening effects of DPC are attributed to its high levels of reducing sugars and phenolics, which intensify Maillard and oxidative browning during baking. This observation is supported by Al-Moalem (2022), and Jahan et al. (2023), who reported that date-seed-enriched breads developed chocolate-like coloration at higher inclusion levels due to enhanced browning reactions. Meanwhile, the lighter color profile of DPSO can be explained by its low pigmentation and lack of browning precursors, which makes it an ideal fat substitute in formulations where color preservation is essential.

#### 3.6.3 Sensory evaluation

Sensory analysis confirmed that DPC and DPSO influenced key sensory attributes of the fortified cakes (Table 5). Attributes assessed included appearance, crust and crumb color, texture, taste, odor, and overall acceptability using a 10-point hedonic scale. Moderate DPC inclusion (12.5 %) maintained consumer acceptability, while higher levels (25–50 %) resulted in significant sensory deterioration (*p* < 0.05). DPSO, by contrast, maintained high acceptability scores across all substitution levels.

**Table 5:** Sensory Evaluation Scores of Fortified and Control Cake Samples.

Sample	Appearance	Crust Color	Crumb Color	Texture	Taste	Odor	Overall Acceptability
Negative Control	10.0 ± 0.0 ^a^	10.0 ± 0.0 ^a^	10.0 ± 0.0 ^a^	9.7 ± 0.0 ^a^	9.5 ± 0.0 ^ab^	9.6 ± 0.0 ^a^	9.6 ± 0.0 ^a^
Positive Control	8.8 ± 1.8 ^ab^	8.6 ± 1.2 ^ab^	9.3 ± 0.9 ^a^	8.7 ± 1.4 ^ab^	9.0 ± 1.1 ^a^	9.2 ± 1.0 ^a^	8.8 ± 1.3 ^a^
DPC-12.5	9.2 ± 1.2 ^ab^	8.7 ± 1.3 ^ab^	9.1 ± 1.2 ^a^	9.0 ± 1.1 ^ab^	9.2 ± 1.0 ^ab^	9.0 ± 1.3 ^a^	9.1 ± 1.2 ^a^
DPC-25	7.5 ± 2.3 ^bc^	7.4 ± 2.5 ^bc^	7.1 ± 2.4 ^b^	7.3 ± 2.2 ^cd^	7.5 ± 2.0 ^c^	7.2 ± 2.3 ^ab^	7.0 ± 2.5 ^b^
DPC-50	6.5 ± 3.0 ^c^	6.2 ± 2.8 ^c^	6.0 ± 3.1 ^b^	5.8 ± 3.5 ^d^	6.3 ± 2.8 ^d^	5.9 ± 3.2 ^b^	5.7 ± 3.4 ^c^
DSO-25	8.9 ± 1.2 ^ab^	9.1 ± 1.1 ^ab^	9.6 ± 1.0 ^a^	9.1 ± 1.2 ^ab^	9.2 ± 1.1 ^ab^	9.6 ± 1.0 ^a^	9.7 ± 1.1 ^a^
DSO-50	8.7 ± 1.5 ^ab^	9.0 ± 1.2 ^ab^	9.3 ± 1.3 ^a^	8.7 ± 1.6 ^abc^	8.7 ± 1.3 ^abc^	9.3 ± 1.2 ^a^	9.2 ± 1.3 ^a^
DSO-100	8.7 ± 1.8 ^ab^	8.8 ± 1.9 ^ab^	9.0 ± 1.6 ^a^	7.9 ± 2.0 ^bc^	8.0 ± 1.7 ^bc^	9.1 ± 1.8 ^a^	8.6 ± 1.9 ^ab^

Values are expressed as mean ± SD (*n* = 3). Different letters indicate statistically significant differences among treatments within each assay (*p* < 0.05).

Appearance scores declined significantly with increasing DPC. While the control and DPC-12.5 scored 10.0 and 9.2, respectively, DPC-25 and DPC-50 dropped to 7.5 and 6.5 (*p* < 0.05), likely due to darker color and denser structure. DPSO-fortified cakes maintained scores of 8.7–8.9, with no significant difference from the control (Jahan et al., 2023; Karambakhsh et al., 2024; Yunusa et al., 2025).

Crust and crumb color preferences reflected instrumental color data: DPC-50 scored lowest (6.2 crust, 6.0 crumb), whereas DPC-12.5 (8.7 and 9.1) was comparable to control. DPSO cakes preserved the golden color expected of pound cake, with crumb color scores exceeding 9.0 even at 100 % substitution (Siddiqui et al., 2000; Frontiers in Sustainable Food Systems, 2023).

Texture ratings were strongly affected by DPC: DPC-50 and DPC-25 scored 5.8 and 7.3, respectively (*p* < 0.05), due to increased fiber density and dryness. In contrast, DPC-12.5 scored 9.0, similar to control (9.7). DPSO cakes had consistently high texture scores, with DPSO-25 reaching 9.1 and DPSO-100 maintaining 7.9 control (Jahan et al., 2023; Karambakhsh et al., 2024; Yunusa et al., 2025). These sensory trends align with instrumental texture data, where DPC increased hardness and gumminess while lowering cohesiveness, effects also documented in sponge-cake studies where higher DPC inclusion led to firmer, less elastic crumbs (Karambakhsh et al., 2024).

Taste followed the same trend. High DPC levels were associated with reduced sweetness and possible bitterness (DPC-50 scored 6.3), whereas DPC-12.5 scored 9.2, comparable to control. DPSO samples had taste scores from 8.0 to 9.2, reflecting the oil's neutral, pleasant flavor and lack of off-notes (Najjar et al., 2022).

Odor scores were highest in the control and DPSO-25 (9.6), while DPC-50 had the lowest at 5.9. DPSO-100 maintained a score of 9.1, confirming that date seed oil did not negatively impact cake aroma (Alkhalidy et al., 2023).

Overall acceptability scores mirrored these findings. DPC-12.5 scored 9.1 (vs. control 9.6), but DPC-50 fell to 5.7. DPSO-25 had the highest overall score (9.7), while DPSO-100 remained favorable at 8.6. A correlation analysis showed that texture (r ≈ 0.85) and taste (r ≈ 0.81) were the strongest predictors of overall liking, whereas color attributes had weaker influence — in line with previous sensory evaluations of fiber-enriched or fat-substituted baked goods (Majzoobi et al., 2018; Yunusa et al., 2025). Previous report also indicate that moderate DPC inclusion (∼12.5–20 %) can provide nutritional enrichment while maintaining acceptable sensory quality, whereas higher substitution compromises texture and overall liking (Karambakhsh et al., 2024).

### 3.7 Shelf-life stability

**Fig. 2:**
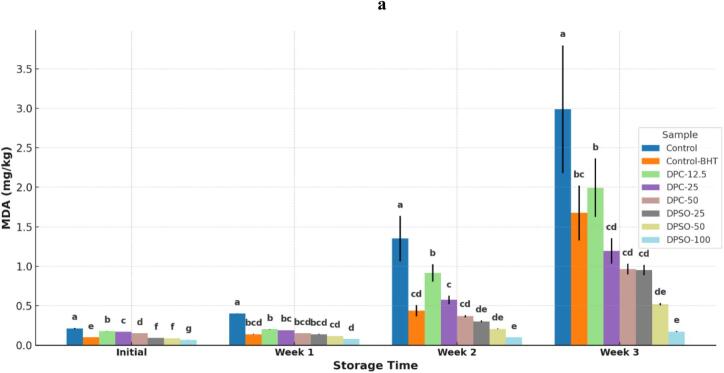

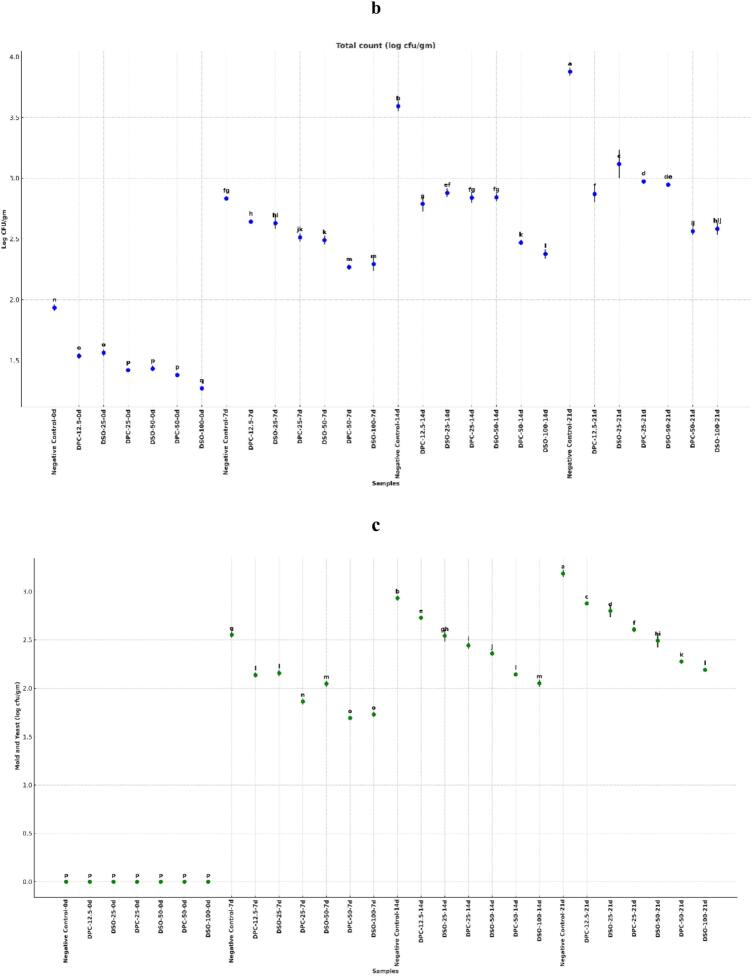
Effect of fortification on (a) malondialdehyde (MDA) levels, (b) total bacterial count, and (c) mold and yeast counts in cake samples during 21 days of storage at ambient temperature. Different letters above the bars at each time point indicate significant differences among treatments (*p* < 0.05).

To evaluate shelf-life stability, two key parameters were assessed over 21 days of ambient storage: lipid oxidation and microbial growth (Fig. 2). The objective was to determine how fortification with DPC and DPSO influenced resistance to rancidity and microbial spoilage—critical indicators of product freshness and safety.

#### 3.7.1 Lipid oxidation

Lipid oxidation was monitored via malondialdehyde (MDA) accumulation using the TBARS assay over 21 days of ambient storage (Fig. 2a). The unfortified control exhibited a sharp rise in MDA, reaching 2.990 mg/kg by day 21, indicating substantial oxidative degradation. In contrast, the BHT-supplemented positive control showed significantly lower MDA levels (1.675 mg/kg, *p* < 0.05), validating its protective effect.

Fortification with DPC and DPSO significantly reduced lipid oxidation in a concentration-dependent manner. DPC-50 and DPSO-100 were the most effective, achieving final MDA levels of 0.964 mg/kg and 0.172 mg/kg, respectively—representing ∼68 % and ∼ 94 % reductions compared to the control. Notably, DPSO-100 outperformed BHT (*p* < 0.05), highlighting its strong oxidative resistance.

Intermediate fortification levels (DPC-25 and DPSO-50) also provided notable protection, reducing MDA by 32–55 %, while DPC-12.5 and DPSO-25 showed moderate yet significant effects (*p* < 0.05).

At intermediate levels, DPC-25 and DPSO-50 also reduced MDA significantly, with estimated reductions of ∼32–55 %, while DPC-12.5 and DPSO-25 were less protective but still conferred statistically significant improvements over the control (*p* < 0.05).

Mechanistically, DPC's protective capacity is primarily attributed to its polyphenol content—especially catechin, quercetin, and chlorogenic acid—which scavenge lipid radicals and chelate transition metals. These findings align with prior studies reporting enhanced lipid stability in bread and rice cakes fortified with date seed derivatives (Jahan et al., 2023; Yunusa et al., 2025).

However, DPC's antioxidant action appeared to plateau at higher inclusion levels, possibly due to limited lipid-phase dispersion or residual oxidizable fats in the formulation. In contrast, DPSO demonstrated superior oxidative suppression, consistent with its high oleic acid content, moderate PUFA levels, and the presence of lipid-soluble antioxidants such as α- and γ-tocopherols and tocotrienols (Al-Khalili et al., 2023).

DPSO's performance may also be attributed to its hydrophobicity, which restricts oxygen diffusion and slows radical propagation. The exceptionally low MDA level in DPSO-100 (0.172 mg/kg) supports this interpretation and is in line with previous findings on the high oxidative stability index of DPSO. I this context, Guo et al. (2020) demonstrated that α-linolenic acid is highly thermolabile, undergoing cis–trans isomerization and degradation under heat. Such transformations reduce lipid quality, but the synergistic presence of tocopherols and oleic acid in DPSO likely mitigates these effects, thereby stabilizing unsaturated fatty acids, including linolenic acid, during baking and storage.

#### 3.7.2 Microbiological stability

Microbial stability during 21-day ambient storage was assessed by tracking total aerobic mesophilic bacteria (Fig. 2b) and mold/yeast counts (Fig. 2c). All samples were microbiologically sterile at day 0. By day 21, the control cake exhibited the highest spoilage levels, reaching 3.88 log CFU/g for bacteria and 3.19 log CFU/g for fungi. In contrast, all DPC- and DPSO-fortified cakes showed significantly lower microbial loads (*p* < 0.05), confirming the shelf-life benefits of both additives.

Among DPC treatments, DPC-50 achieved the greatest microbial suppression, with 2.56 log CFU/g bacteria (33.9 % reduction) and 2.28 log CFU/g molds/yeasts (35.4 % reduction). DPC-25 and DPC-12.5 also reduced microbial counts in a concentration-dependent manner. Similar trends were observed with DPSO; DPSO-100 reached 2.58 log CFU/g for bacteria (33.4 % reduction) and 2.19 log CFU/g for fungi (34.2 % reduction), matching DPC-50 in efficacy.

The antimicrobial action of DPC is likely attributed to its phenolic constituents—catechin, quercetin, chlorogenic acid, ellagic acid—and tannins, all known for disrupting microbial membranes, interfering with enzymes, and chelating metal ions (El Sohaimy et al., 2015; Zihad et al., 2021). These results are consistent with Jahan et al. (2023), who showed that breads fortified with 5–10 % date seed powder exhibited delayed microbial spoilage, remaining acceptable for up to 6 days compared to only 3 days for the control. This highlights the role of date seed phenolics and fiber in prolonging microbiological stability in bakery matrices. Similarly, Yunusa et al. (2025) reported microbial suppression in rice cakes fortified with date seed derivatives.

DPSO's antimicrobial effect is likely mediated by its lipid-phase components. Fatty acids such as lauric and oleic acid, along with α- and γ-tocopherols, contribute to microbial inhibition by disrupting membranes and preventing oxidation. Its hydrophobic matrix may also limit moisture and oxygen diffusion, creating a less favorable surface for microbial colonization (Al-Khalili et al., 2023).

These findings extend previous reports on the antimicrobial properties of date extracts, where Ouamnina et al. (2024) showed that phenolic- and tannin-rich fractions disrupted microbial membranes and inhibited enzymatic activity. In the present study, the whole-matrix incorporation of DPC and DPSO into baked products produced a similar outcome, effectively reducing microbial spoilage. The comparable efficacy of DPC-50 and DPSO-100 to synthetic antioxidants positions them as promising clean-label alternatives for microbial control in bakery systems.

Taken together, the lipid oxidation and microbial growth data indicate that fortification with DPC or DPSO effectively extended the shelf-life of cakes from less than one week in the control to at least 21 days under ambient storage.

### 3.8 Overall evaluation of samples

**Fig. 3:**
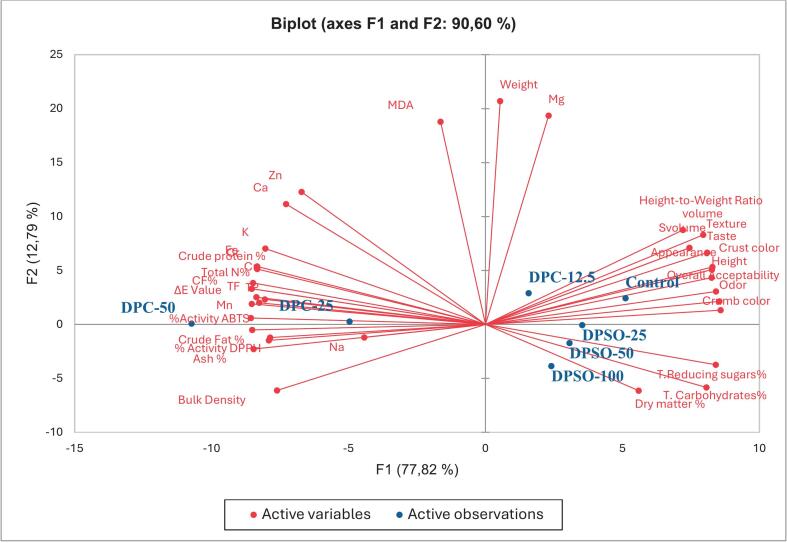
Principal component analysis (PCA) biplot illustrating the distribution of cake samples fortified with date press cake and date seed oil based on compositional, technological, antioxidant, and sensory attributes.

Principal component analysis (PCA) was used to interpret the multivariate relationships among compositional, functional, oxidative, and sensory attributes of cakes fortified with DPC and DPSO (Fig. 3). The two principal components accounted for 90.60 % of the total variance (F1: 77.82 %, F2: 12.79 %), with the first component primarily separating treatments based on bioactive content, physical performance, and sensory acceptance. The strong positive loading of TP, TF, and antioxidant capacity variables on F1 indicates that phenolic retention was a dominant factor influencing sample differentiation. In particular, the location of DPC-fortified cakes along F1 reflects the contribution of catechin, chlorogenic acid, and related compounds to both oxidative stability and microbial suppression.

DPC-12.5 and DPC-25 clustered with TP, TF, and dietary fiber, confirming their shared contribution to antioxidant performance and structural quality. These results reflect the well-documented interactions between insoluble polysaccharides (such as cellulose and lignin) and polyphenols like catechin (2129.1 μg/g) and chlorogenic acid (781.2 μg/g), which stabilize phenolics during thermal processing through hydrogen bonding and hydrophobic association (Das et al., 2023). Their retention was confirmed by LC–MS and reflected in the proximity of these treatments to DPPH and ABTS vectors, indicating redox activity was preserved in the baked matrix.

The antioxidant role of these compounds extended beyond radical scavenging. Catechin disrupts bacterial membranes via interactions with phospholipid headgroups, while chlorogenic acid inhibits ATPase activity in Gram-positive organisms such as *Bacillus* (El Sohaimy et al., 2015; Zihad et al., 2021). Consequently, DPC-12.5 and DPC-25 reduced total microbial counts by 30–35 % after 21 days of storage compared to the control, offering in situ evidence of antimicrobial activity.

DPC-50, situated on the far left of F1, aligned with ash, bulk density, and minerals including Fe and Mn. Despite its elevated phenolic and mineral content, this treatment showed reduced sensory quality, a likely consequence of gluten dilution, darker color, and fiber-induced grittiness*.* Nonetheless, oxidative stability remained high, with MDA levels 68 % lower than the control. This may be attributed to a dual mechanism: (1) catechol-containing phenolics chelate Fe^3+^ (log K ≈ 8.5), disrupting hydroxyl radical formation through inhibition of Fenton reactions (Perron et al., 2010), and (2) co-existing phenolics sequester Fe and Mn, reducing microbial access to these essential cofactors (Prakash et al., 2022). This phenolic–mineral matrix likely accounts for DPC-50's ability to suppress microbial growth despite its higher reducing sugar content (25.9 %), which could otherwise support fermentation.

DPSO treatments (DSO-25 to DSO-100) clustered with technological and sensory quality vectors, including moisture retention, crumb softness, and color traits. Although DPSO lacks a broad phenolic spectrum, it contributed to shelf-life extension through lipid-phase protection. α- and γ-tocopherols in the oil terminate lipid peroxidation by donating hydrogen atoms to lipid peroxyl radicals (LOO•), forming stable, non-propagating tocopheryl radicals (Alkhalidy et al., 2023). This lipid-phase protection was not captured by DPPH or ABTS assays but was reflected in the TBARS results: DPSO-100 reduced MDA by 94 %, outperforming BHT-supplemented controls.

In addition to oxidative stability, the oleic acid content of DPSO (115.2 μg/g) may have contributed to microbial inhibition by altering bacterial membrane fluidity, weakening lipid bilayers and increasing permeability—an effect particularly pronounced against *Pseudomonas* spp. (Obukhova & Murzina, 2024). This mechanism is particularly effective against spoilage bacteria such as *Pseudomonas*. Tocopherols may also interfere with bacterial quorum sensing by degrading acyl-homoserine lactones, delaying biofilm formation (Hamza et al., 2021; Obukhova & Murzina, 2024). These effects likely explain DPSO-100's low microbial counts (2.19 log CFU/g) after storage.

Overall, PCA confirms the complementary roles of DPC and DPSO in cake fortification. DPC enhances antioxidant capacity, fiber content, and mineral density but requires dosage control to avoid textural and sensory drawbacks. DPSO supports lipid stabilization, moisture retention, and microbial resistance through non-phenolic antioxidants and favorable lipid composition. The combination of DPC-12.5 and DPSO-50 offers a balanced strategy for developing clean-label cakes with improved nutritional, functional, and storage characteristics.

## 4 Conclusion

Date press cake and date seed oil demonstrated complementary roles as sustainable, multifunctional fortifiers in cake systems. Raw DPC significantly enhanced the nutritional profile, contributing dietary fiber, minerals (Fe, Zn, Mn), and high levels of phenolics and flavonoids. When incorporated into cakes, DPC-50 achieved the greatest enrichment (548 mg GAE and 87 mg QE per 100 g), translating into improved antioxidant capacity and microbial inhibition, though with reduced sensory appeal. In contrast, lower inclusion (DPC-12.5) maintained desirable texture while still offering functional benefits.

LC–MS profiling confirmed the retention of key thermally stable phenolics such as catechin and chlorogenic acid, supporting both radical scavenging and antimicrobial effects. Although DPSO contained minimal hydrophilic phenolics, it provided lipid-phase protection via tocopherols and unsaturated fatty acids, with DPSO-100 reducing MDA by 94 % and lowering microbial counts while maintaining sensory acceptability.

Overall, DPC and DPSO provided complementary benefits: DPC enhanced aqueous-phase antioxidant and antimicrobial activity, while DPSO contributed to lipid stability and microbial control. Nevertheless, this study has some limitations, including the use of in vitro assays that may not fully reflect in vivo digestion, the potential underestimation of bound or thermally modified phenolics by colorimetric methods, the relatively small sensory panel size, and the lack of long-term dietary assessment. These aspects should be addressed in future studies to confirm applicability in real-world bakery production. Their incorporation supports clean-label product development and valorization of date by-products within a circular economy framework.

## CRediT authorship contribution statement

**Abdelrahman R. Ahmed:** Writing – review & editing, Writing – original draft, Visualization, Supervision, Software, Funding acquisition, Formal analysis, Conceptualization. **Khaled M.A. Ramadan:** Writing – review & editing, Writing – original draft, Supervision, Software, Methodology, Investigation, Conceptualization. **Haiam O. Elkatry:** Writing – review & editing, Supervision, Methodology, Conceptualization. **Nashi K. Alqahtani:** Writing – review & editing, Supervision, Funding acquisition. **Eslam S.A. Bendary:** Writing – review & editing, Investigation, Formal analysis. **Mahmoud M. Ghuniem:** Writing – review & editing, Methodology, Investigation, Formal analysis. **Mohamed A.A. Mahmoud:** Writing – review & editing, Writing – original draft, Visualization, Methodology, Formal analysis, Conceptualization.

## Ethical statement

The study followed the Declaration of Helsinki and King Faisal University safety guidelines (Approval No. KFU-2025-Ethics-3445). Written consent was obtained from all sensory panelists.

## Funding

This research work was supported and funded by theDate Palm Research Center of Excellence, King Faisal University, Saudi Arabia (Project number DPRC-26-2024).

## Declaration of competing interest

The authors declare that they have no known competing financial interests or personal relationships that could have appeared to influence the work reported in this paper.

## Data Availability

Data will be made available on request.

## Appendix A Supplementary data

Supplementary material Supplementary Table 1: Cake Formulations with Different Levels of Date Press Cake (DPC) and Date Seed Oil (DPSO) Substitution; **Supplementary Fig. 1.** Antioxidant activity (DPPH and ABTS assays) of control and fortified cake samples. Results are expressed as mean ± SD (*n* = 3). Different letters indicate significant differences among treatments within each assay (*p* < 0.05); **Supplementary Fig. 2.** Effect of fortification on cake color attributes: (a) crumb color, (b) crust color, and (c) total color difference (*ΔE**) in crumb and crust relative to the negative control. Different letters on bars within each attribute indicate significant differences among treatments (*p* < 0.05). Image 1
