# Supplementary material for: Valorization of date palm by-products (Phoenix dactylifera L.) in cake fortification: Nutritional enrichment, antioxidant retention, and shelf-life extension
Source: Food Chem X. 2025 Oct 17;31:103184. doi: 10.1016/j.fochx.2025.103184 (PMC12554979; doi:10.1016/j.fochx.2025.103184)
Supplement: Supplementary file 1 — Supplementary material Supplementary Table 1: Cake Formulations with Different Levels of Date Press Cake (DPC) and Date Seed Oil (DPSO) Substitution; Supplementary Fig. 1. Antioxidant activity (DPPH and ABTS assays) of control and fortified cake samples. Results are expressed as mean ± SD (n = 3). Different letters indicate significant differences among treatments within each assay (p < 0.05); Supplementary Fig. 2. Effect of fortification on cake color attributes: (a) crumb color, (b) crust color, and (c) total color difference (ΔE*) in crumb and crust relative to the negative control. Different letters on bars within each attribute indicate significant differences among treatments (p < 0.05). [file mmc1.docx]

**Valorization of Date Palm By-Products *(Phoenix dactylifera L.)* in Cake Fortification: Nutritional Enrichment, Antioxidant Retention, and Shelf-Life Extension**

**Abdelrahman R. Ahmed^1^*, Khaled M. A. Ramadan^2^, Haiam O. Elkatry^1^, Nashi K. Alqahtani^1^, Eslam S.A. Bendary^3^, Mahmoud M. Ghuniem^4^, Mohamed A. A. Mahmoud^3^***

**Supplementary Materials**

**Supplementary Table 1** Cake Formulations with Different Levels of Date Press Cake (DPC) and Date Seed Oil (DPSO) Substitution.

| **Ingredients** | **In Grams** | | | | | | | **BHT (ppm)** |
| --- | --- | --- | --- | --- | --- | --- | --- | --- |
|  | **Flour** | **Sugar** | **Shortening** | **Fresh whole egg** | **Dry milk powder** | **Baking powder** | **Vanillin** |  |
| **Negative Control** | 100 | 60 | 50 | 85 | 3 | 4 | 0.6 | 0 |
| **Positive Control** | 100 | 60 | 50 | 85 | 3 | 4 | 0.6 | 200 |
| **DPC-12.5** | 100 | 52.5 | 43.75 | 85 | 3 | 4 | 0.6 | 0 |
| **DPC-25** | 100 | 45 | 37.5 | 85 | 3 | 4 | 0.6 | 0 |
| **DPC-50** | 100 | 30 | 25 | 85 | 3 | 4 | 0.6 | 0 |
| **DPSO-25** | 100 | 60 | 50 | 85 | 3 | 4 | 0.6 | 0 |
| **DPSO-50** | 100 | 60 | 50 | 85 | 3 | 4 | 0.6 | 0 |
| **DPSO-100** | 100 | 60 | 50 | 85 | 3 | 4 | 0.6 | 0 |


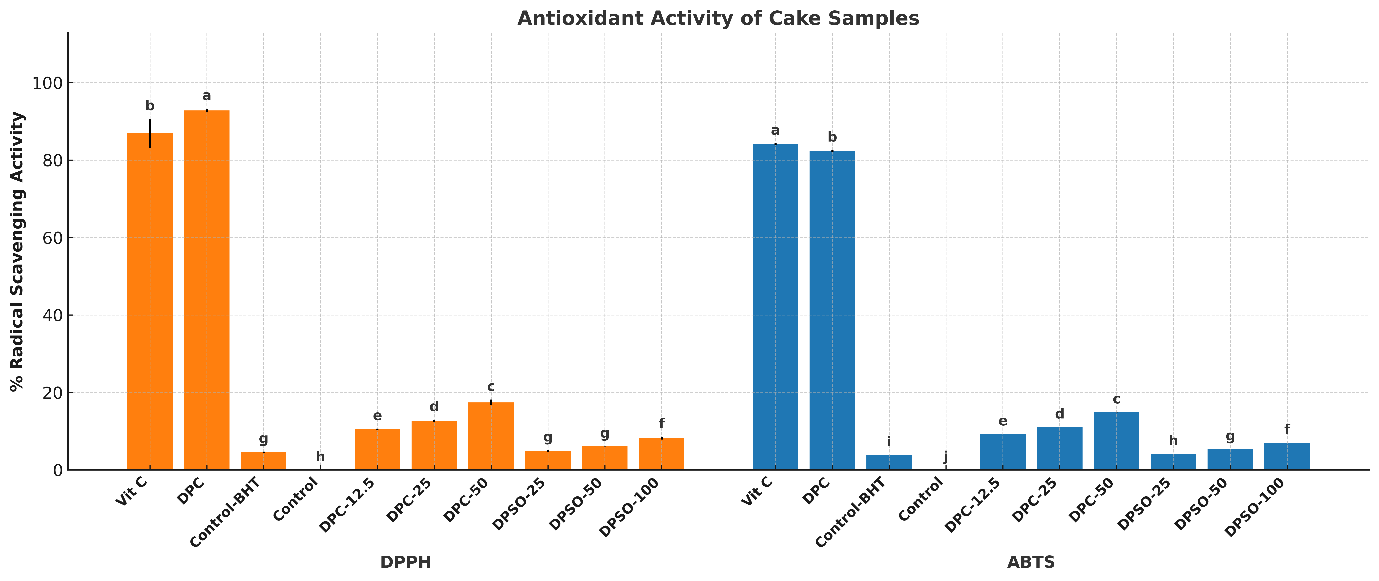


**Supplementary Figure 1.** Antioxidant activity (DPPH and ABTS assays) of control and fortified cake samples. Results are expressed as mean ± SD (*n* = 3). Different letters indicate significant differences among treatments within each assay (*p* < 0.05).

| **a**  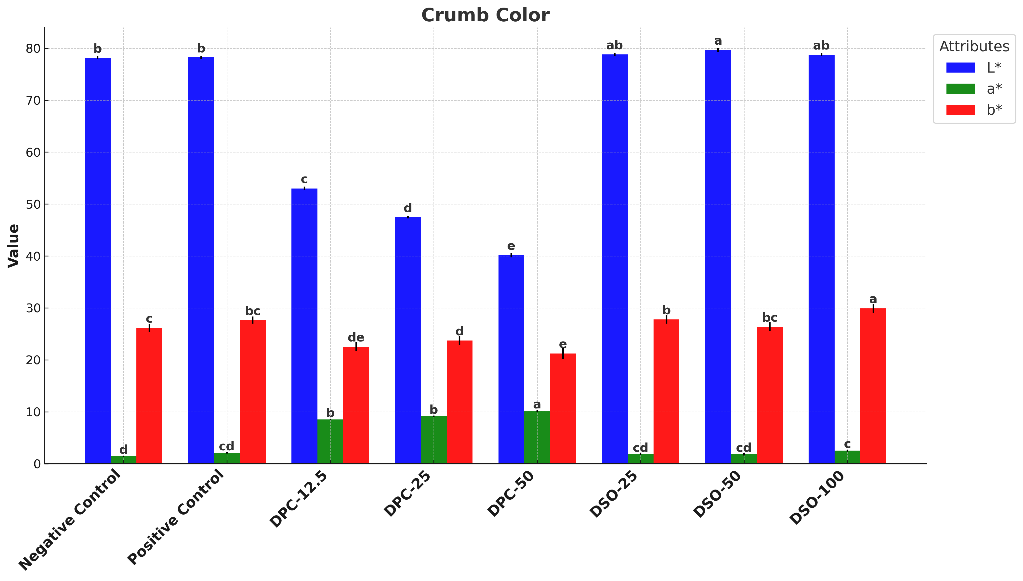 |
| --- |
| **b**  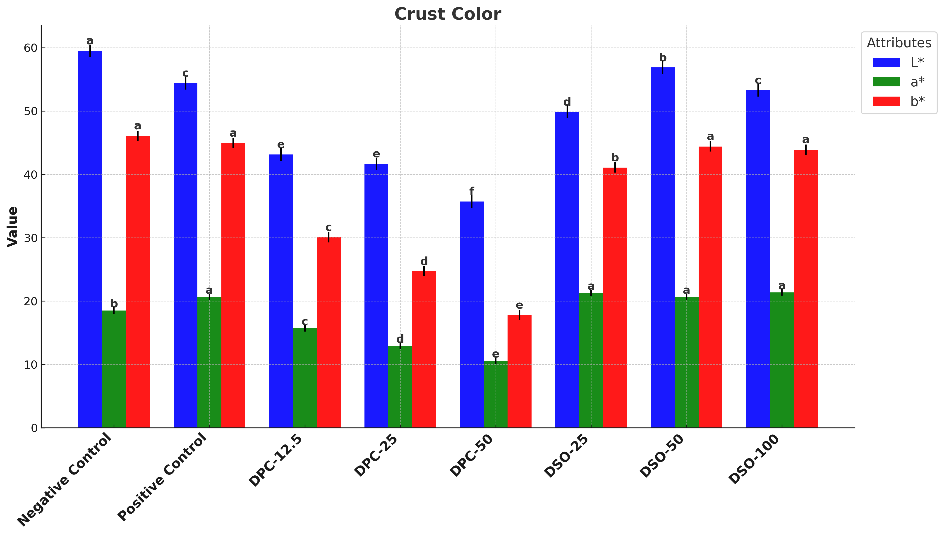 |
| **c**  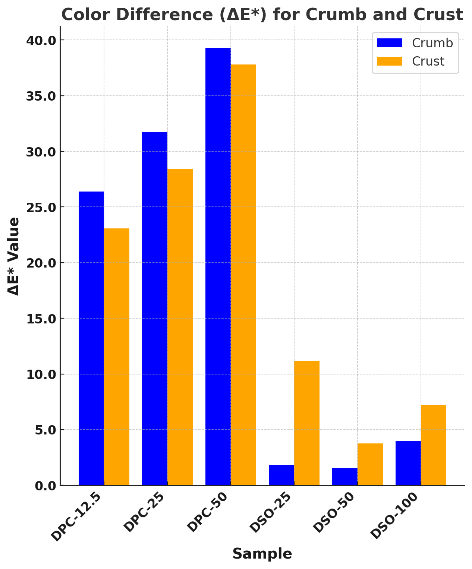 |

**Supplementary Figure 2.** Effect of fortification on cake color attributes: (a) crumb color , (b) crust color, and (c) total color difference (*ΔE**) in crumb and crust relative to the negative control. Different letters on bars within each attribute indicate significant differences among treatments (*p* < 0.05).
